# Analysis of Dielectric Waveguide Grating and Fabry–Perot Modes in Elastic Grating in Optical Detection of Ultrasound

**DOI:** 10.3390/s21124081

**Published:** 2021-06-14

**Authors:** Suejit Pechprasarn, Chayanisa Sukkasem, Phitsini Suvarnaphaet

**Affiliations:** College of Biomedical Engineering, Rangsit University, Pathum Thani 12000, Thailand; suejit.p@rsu.ac.th (S.P.); chayanisa.s61@rsu.ac.th (C.S.)

**Keywords:** optical detection of ultrasound, interferometer, dielectric waveguide grating, instrumentation

## Abstract

In our previous work, we have demonstrated that dielectric elastic grating can support Fabry–Perot modes and provide embedded optical interferometry to measure ultrasonic pressure. The Fabry–Perot modes inside the grating provide an enhancement in sensitivity and figure of merit compared to thin film-based Fabry–Perot structures. Here, in this paper, we propose a theoretical framework to explain that the elastic grating also supports dielectric waveguide grating mode, in which optical grating parameters control the excitation of the two modes. The optical properties of the two modes, including coupling conditions and loss mechanisms, are discussed. The proposed grating has the grating period in micron scale, which is shorter than the wavelength of the incident ultrasound leading to an ultrasonic scattering. The gap regions in the grating allow the elastic grating thickness to be compressed by the incident ultrasound and coupled to a surface acoustic wave mode. The thickness compression can be measured using an embedded interferometer through one of the optical guided modes. The dielectric waveguide grating is a narrow bandpass optical filter enabling an ultrasensitive mode to sense changes in optical displacement. This enhancement in mechanical and optical properties gives rise to a broader detectable pressure range and figure of merit in ultrasonic detection; the detectable pressure range and figure of merit can be enhanced by 2.7 times and 23 times, respectively, compared to conventional Fabry–Perot structures.

## 1. Introduction

Photoacoustic imaging (PI) has been of interest to the science and engineering community because of its complementary capability to optical imaging in measuring mechanical properties of samples, such as Young’s modulus and stiffness [1], rather than the optical properties, such as refractive index, reflectance, and transmittance. PI has proven to be applicable in a wide range of applications in several fields, including material science [2,3], biological science [4], and medical science [5].

PI’s current challenges to obtain high-resolution images are: (1) the generation of high-frequency ultrasound; the resolution depends on the bandwidth (Δ*f*) of the ultrasound. For the conventional 50 MHz sources, the ultrasonic image lateral resolution is around 80 μm to 160 μm, and 20 μm to 100 μm in axial resolution [6,7]. Of course, to increase the resolution, there is a demand for the bandwidth of the ultrasound. Thermal expansion due to femtosecond laser illumination of materials [8] and a thin piezoelectric layer [9] can provide a broadband ultrasonic source. (2) For biological and medical applications, the specimens are usually aqueous, and this is problematic since the penetration depth in the water of the ultrasound at GHz regime is attenuated at the rate of depth square [10]. Therefore, this leads to (3) high demand for ultrasonic sensors’ sensitivity [11].

Optical surfaces and structures have been employed for optical detection of high-frequency ultrasound, including piezoelectric devices [12], fiber-based optical transducers [13], optical ring resonators [14,15], and thin film-based sensors [11]. The piezoelectric device has a tradeoff between the sensitivity and the detection bandwidth. It also requires a long working distance, and it is bulky and not scalable [16]. The ring resonator and the fiber-based sensor have shown a good sensitivity, but they both have a limited detection bandwidth depending on the fabricated sensor’s size [17].

**Figure 1:**
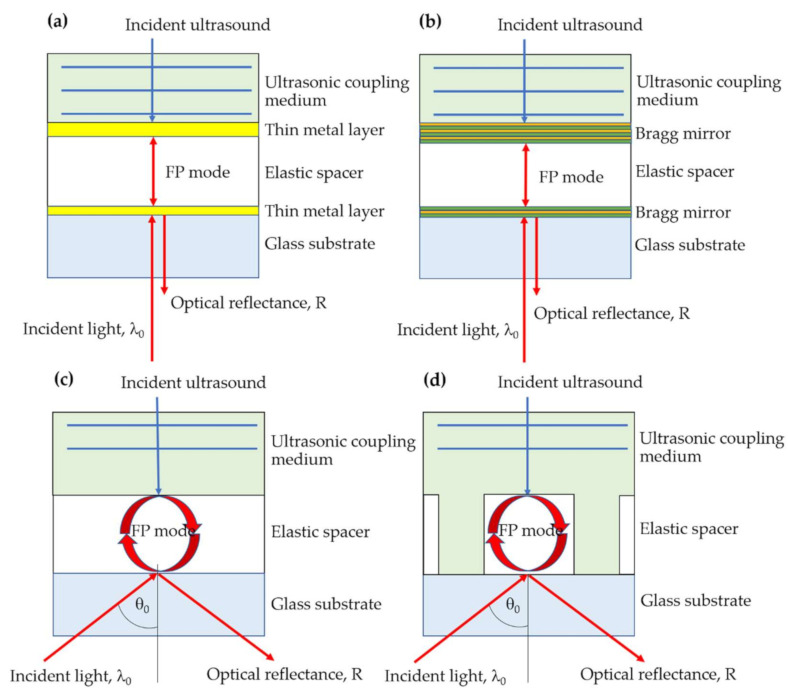
(**a**) Fabry–Perot (FP) mode in bimetallic mirrors, (**b**) FP mode in Bragg reflectors, (**c**) FP mode in uniform polydimethylsiloxane (PDMS), and (**d**) FP mode in PDMS grating.

For the thin film-based technologies, the well-known structures for measuring ultrasound are Fabry–Perot (FP) interferometers [18], including bimetallic layer structure [19,20], Bragg mirrors [21], uniform elastic film [11], surface plasmon resonance [22], and elastic grating structure [23]. These FP structures, as shown in Figure 1, have demonstrated an ultra-sensitivity to incident ultrasound. We have recently proposed a theoretical study to compare the performance of several thin-film technologies [23]. One unique advantage of the transparent thin film-based sensors, such as the uniform polydimethylsiloxane (PDMS) layer [11] and the PDMS grating [23], is that the material transparency has offered a unique advantage to incorporate the ultrasonic detection with conventional optical imaging modalities.

In Sukkasem et al. [23], we have recently demonstrated that a 150-micron size grating period, as shown in Figure 1d, can support the FP mode and provide higher sensitivity and figure of merit (FOM) compared to the other uniform FP structures. Here, we demonstrate that the transparent PDMS thin film gratings in submicron and several micron grating periods can support the FP modes and dielectric waveguide grating mode (DWG) [24]. Phase matching condition [25] and phase cancellation [26] between eigenmodes inside the dielectric grating can form ultrasensitive optical interferometry, sensitive to the incident ultrasound. We provide a theoretical framework to explain how an incident light can be coupled to the DWG mode and discuss the loss mechanism explaining how the DWG mode can measure the incident ultrasonic wave pressure. The DWG mode in an elastic grating structure for ultrasonic detection has never been studied and reported before in the literature to the best of the authors’ knowledge.

## 2. Materials and Methods

### 2.1. Mechanical Simulation Using Finite Element Method

**Figure 2:**
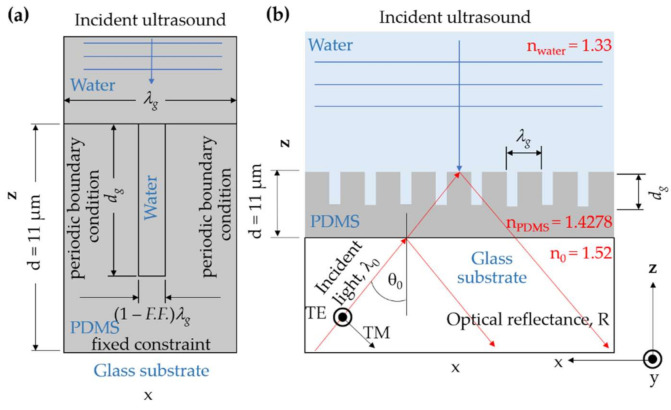
(**a**) The finite element method (FEM) model of the PDMS grating in COMSOL, and (**b**) optical detection scheme for measuring the incoming ultrasound.

The finite element method (FEM) using COMSOL Multiphysics 5.3a was employed to compute mechanical and structure deformation responses due to ultrasonic loading on the PDMS grating, as shown in Figure 2a. The FEM calculation employed the acoustic-solid interaction model. There was a continuous ultrasound source radiating at 2 MHz frequency on the top of the FEM model. The ultrasound then propagated through the water coupling medium before compressing the PDMS grating. The material compression can be calculated by solving the Helmholtz equation [27] and Navier’s equations [28]. The other boundary conditions of the FEM model included the left and right edges of the FEM model and were set to the periodic boundary condition, which was essentially Floquet–Bloch theory [29,30]. The bottom of the model was stationary using a fixed constraint. All the FEM simulations reported in this paper were computed with the mesh size of 15 nm, ensuring that the models have reached their convergence.

The PDMS is one of the highly elastic and viscoelastic materials. It has been well established that the PDMS’s hyperplastic properties are negligible [31]. The Young’s modulus (*E*) of PDMS [31] is 123.4 MPa [11], and Poisson’s ratio is 0.43 [31] at 2 MHz ultrasonic frequency and 11 μm PDMS film thickness (*d*). Young’s modulus depends on the ultrasonic frequency loading and the thickness of the PDMS layer [23]. Here we adopted Young’s modulus and the Poisson’s ratio for 11 μm thick PDMS and 2 MHz ultrasonic frequency, reported and experimentally verified in the literature [11]. The 2 MHz frequency is a standard medical ultrasound imaging frequency and is usually used to test and characterize ultrasonic sensors [32]. This frequency allows a direct comparison between different ultrasonic sensing platforms reported in the literature [11,23]. The other grating parameters are defined as depicted in Figure 2a; grating period (*λ_g_*), depth of the grating groove (*d_g_*), grating fill factor (*F.F.*), and the aspect ratio is defined as *F.F. λ_g_/d_g_*.

### 2.2. Optical Simulation Using Rigorous Coupled-Wave Theory

Rigorous coupled-wave analysis (RCWA) [33] software has been implemented under MATLAB R2021a, utilizing parallel computing and graphic processing unit (GPU) computing. It is employed to calculate reflection coefficients and reflectance from the PDMS grating when the grating is illuminated by a coherent optical wavelength (*λ*_0_) of 685 nm at the incident angle (*θ*_0_), as depicted in Figure 2b.

There are two polarization directions: the transverse electric (TE) and the transverse magnetic (TM) were investigated in this study. The incident light is coupled through a glass prism and the matching oil with a refractive index (*n*_0_) of 1.52. The PDMS refractive index (*n_PDMS_*) is 1.4278 [34]. All RCWA calculations computed with 101 diffracted orders covered higher-order eigenmodes’ effects and achieved simulation convergence.

When the ultrasound is illuminated on the top of the grating, as shown in Figure 2b, the grating is compressed, leading to the change in the grating’s PDMS thickness, which can be calculated using the FEM model the protocol described in Section 2.1. The change in the thickness introduces the change in optical reflectance, which can be computed using RCWA as described in Section 2.2. However, when the external force compresses an elastic material, the stress accumulated inside the material leads to local accumulative stress and local refractive index change, it is established and validated that the effect of the local refractive index change is negligible compared to the change in the thickness [11,18]. The refractive index of the PDMS was fixed at a constant value of 1.4278 when illuminated with the ultrasound. 

### 2.3. Ultrasonic Sensing Performance Parameters

Performance parameters employed in this manuscript were adopted from our previous publication [23] to compare the proposed DWG mode with reported structures in the literature.

Sensitivity (S) defined as the change in optical reflectance over the pressure of ultrasonic loading as expressed in Equation (1):

(1) $$S=\left|\frac{R_{L,0}-R_{L,U}}{P}\right|$$

where *S* is the sensitivity in Pa^−1^. $R_{L,0}$ and $R_{L,U}$ are linearized reflectance with no ultrasonic loading and with the incident ultrasonic pressure (*P*). Note that sensorgrams of the proposed grating were linearized using the polynomial equation of degree three, as described in details in Sukkhasem et al. [23]:

*FOM* is defined as the sensitivity over the $R_{L,0}$.

Detectable pressure range (α) is defined as the acoustic pressure range that the sensor output can respond linearly as a function of varying ultrasonic pressures [23].

## 3. Results and Discussion 

### 3.1. Coupling of Ultrasonic Modes in the Polydimethylsiloxane (PDMS) Grating

**Figure 3:**
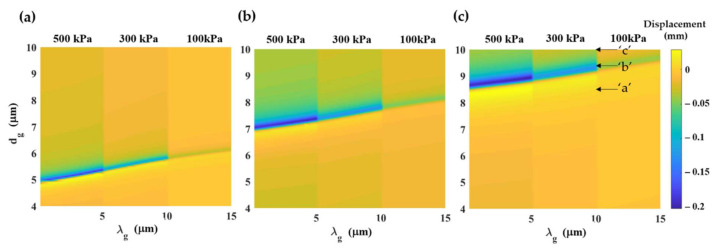
Grating thickness compression in μm calculated using the FEM simulation for different grating structures with *d* of 11 μm with a varying grating thickness (shown in y-axis) from 4 μm to 11 μm and a varying grating period (shown in x-axis) from 0 μm to 15 μm, and (**a**) *F.F.* of 0.99, (**b**) *F.F.* of 0.98, and (**c**) *F.F.* of 0.97 under 2 MHz ultrasonic pressures of 500 kPa, 300 kPa, and 100 kPa.

Figure 3 shows a contour map of PDMS grating compressions for different grating aspect ratios, grating periods, and fill factors when the structure thickness *d* is fixed at 11 μm and the 2 MHz incident ultrasound pressure of 100 kPa, 300 kPa, and 500 kPa. The incident ultrasound can significantly compress some gratings, and this is due to the surface acoustic wave (SAW) mode coupling [23], which depends on the grating parameters and the ultrasonic incident frequency. The relationship of grating parameters that can couple the incident ultrasound into the SAW mode is given in Equation (2).

(2) $$d_{g}=0.08\times \lambda_{g}-2500\times {\left(1-FF\right)}^{2}+280\times (1-FF)+2.4$$

**Figure 4:**
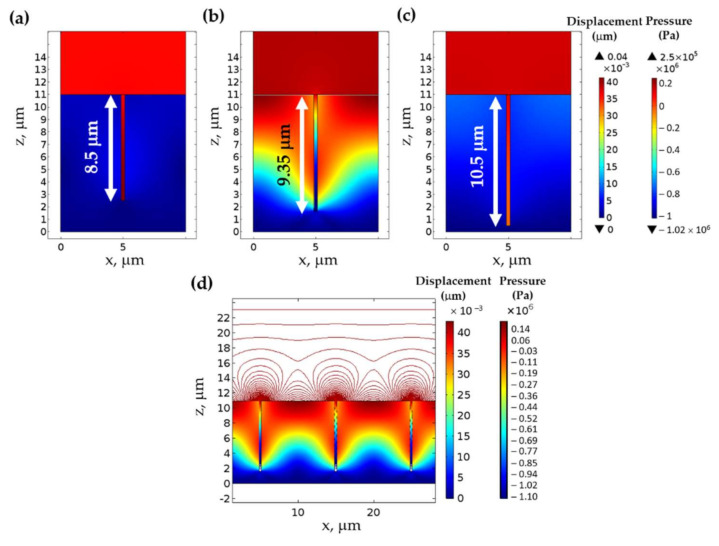
Pressure contours in Pa and grating structure compression in μm calculated using the FEM simulation for the gratings with *λ_g_* of 10 μm, *F.F.* of 0.97, *d* of 11 μm, and different grating heights *d_g_* when illuminated with 100 kPa of 2 MHz ultrasound. (**a**) *d_g_* of 8.5 μm, (**b**) *d_g_* of 9.35 μm, and (**c**) *d_g_* of 10.5 μm, and (**d**) contour pressure level and grating structure compression for the *d_g_* of 9.35 μm. Note that the left color bar shows the magnitude of the grating structure compression in μm, and the right color bar shows the pressure level in Pa.

Figure 4 shows the pressure contour map of the three grating structures labeled ‘a’, ‘b’, and ‘c’ in Figure 3. The three structures have the same size of the grating period *λ_g_* of 10 μm and fill factor *F.F.* of 0.97. The three gratings had different groove depth *d_g_* of 8.5 μm, 9.35 μm, and 10.5 μm for the gratings ‘a’, ‘b’, and ‘c’. Figure 4a,c shows the gratings ‘a’ and ‘c’ had less thickness compression than the grating ‘b’, as shown in Figure 4b. The grating ‘b’ had a standing wave pattern of the ultrasound intensity on the grating surface due to the SAW mode coupling, as shown in Figure 4d. There is a linear relationship between the incident ultrasonic pressure and the PDMS compression. For the SAW structure, the compression is 4.10 × 10^−13^ m/Pa; meanwhile, for the non-SAW structure, the compression is 9.38 × 10^−14^ m/Pa. These SAW modes also occur in other grating regimes, such as larger *F.F.* and longer *λ_g_* regimes, as recently reported in Sukkasem et al. [23]. Here, the gratings with *d* of 11 μm were chosen to directly compare with the performance of the other thin film-based structures reported in Sukkasem et al. [23] and Learkthanakhachon et al. [11].

### 3.2. Optical Responses of the PDMS Grating

**Figure 5:**
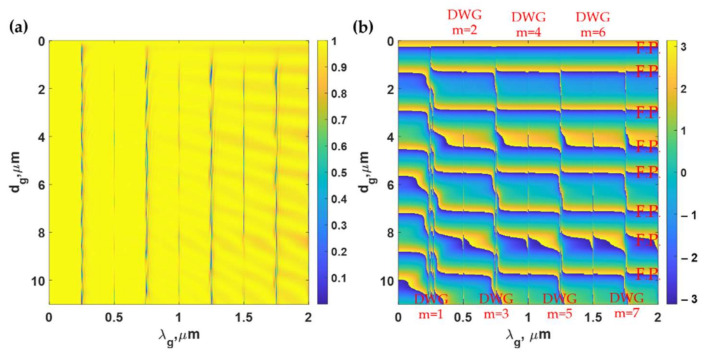
(**a**) Reflectance and (**b**) phase responses in rad of the reflection coefficients calculated using rigorous coupled-wave analysis (RCWA) for PDMS gratings when the grating period *λ_g_* was varied from 0 μm to 2 μm, and the grating groove depth *d_g_* was varied from 0 μm (uniform PDMS layer 11 μm thick) to 11 μm when illuminated by transverse magnetic (TM) polarized light at 685 nm and the *n*_0_*sinθ*_0_ of 1.37.

**Figure 6:**
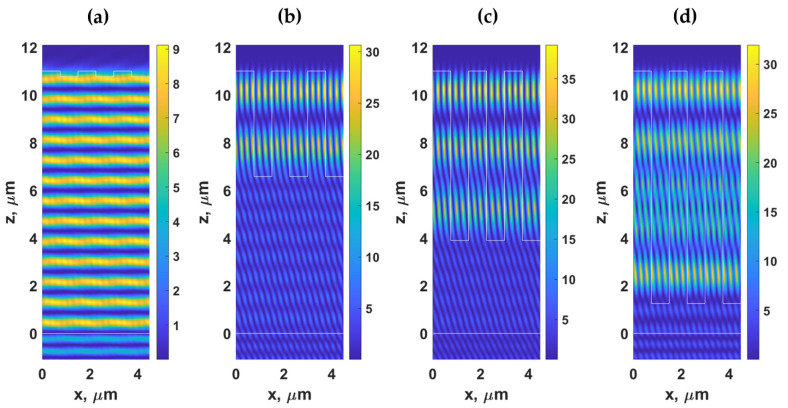
$H_{y}^{2}$ field distribution calculated using RCWA for the gratings with *λ_g_* of 1.5 μm, *F.F.* of 0.5, *d* of 11 μm, and (**a**) *d_g_* of 0.24 μm, (**b**) *d_g_* of 4.42 μm, (**c**) *d_g_* of 7.11 μm, and (**d**) *d_g_* of 9.75 μm, when illuminated by TM polarized light at 685 nm and the *n*_0_*sinθ*_0_ of 1.37.

For the optical response of the PDMS grating, the incident angle was fixed at *n*_0_*sinθ*_0_ of 1.37, illuminating the PDMS grating with the TM polarized light at the wavelength of *λ*_0_ of 685 nm. Figure 5a,b shows reflectance and the optical phase responses of the reflection coefficients of PDMS gratings when the gratings period *λ_g_* were varied from 0 μm to 2 μm and the grating groove depth *d_g_* from 0 μm (uniform PDMS layer 11 μm thick) to 11 μm, and the *F.F.* of 0.5. The phase profile in Figure 5b shows two main modes in these gratings, which are (1) vertical modes occurring at *λ_g_* of 0.25 μm, 0.5 μm, 0.75 μm, 1 μm, 1.25 μm, 1.5 μm, 1.75 μm, and 2 μm respectively labeled as ‘DWG’ in Figure 5b, and (2) the FP modes in the horizontal lines labeled as ‘FP’. The FP resonances are supported by both the uniform PDMS layer and the grating, as illustrated by the y-axis magnetic intensity $H_{y}^{2}$ in Figure 6. Note that the results for TE polarization were similar to the TM polarization; they are, therefore, omitted to save space.

**Figure 7:**
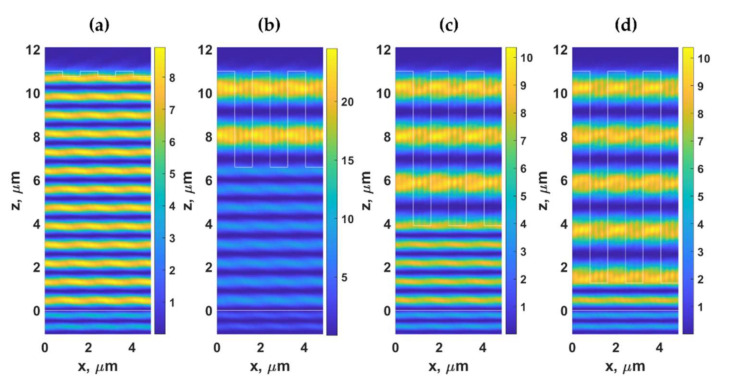
$H_{y}^{2}$ field distribution calculated using RCWA for the gratings with *λ_g_* of 1.625 μm, *F.F.* of 0.5, *d* of 11 μm, and (**a**) *d_g_* of 0.24 μm, (**b**) *d_g_* of 4.42 μm, (**c**) *d_g_* of 7.11 μm, and (**d**) *d_g_* of 9.75 μm, when illuminated by TM polarized light at 685 nm and the *n*_0_*sinθ*_0_ of 1.37.

Figure 7a–d show the $H_{y}^{2}$ field distribution of the gratings with *λ_g_* of 1.625 μm, *F.F.* of 0.5, *d* of 11 μm, and *d_g_* of 0.24 μm, *d_g_* of 4.42 μm, *d_g_* of 7.11 μm, and *d_g_* of 9.75 μm, respectively, when illuminated by TM polarized light at 685 nm and the *n*_0_*sinθ*_0_ of 1.37. The grating period *λ_g_* of 1.625 μm enabled us to suppress the effect of dielectric waveguide grating since the *λ_g_* of 1.625 μm did not support the DWG mode, as shown in Figure 5. Therefore, the field patterns shown in Figure 7 were dominantly the FP modes. The difference between the two modes will be discussed in the next section, explaining how these modes have different energy dissipation mechanisms. The FP distributed their energy mainly through the −1st and 1st diffracted orders, whereas the DWG modes leaked their energy out through higher diffracted orders.

**Figure 8:**
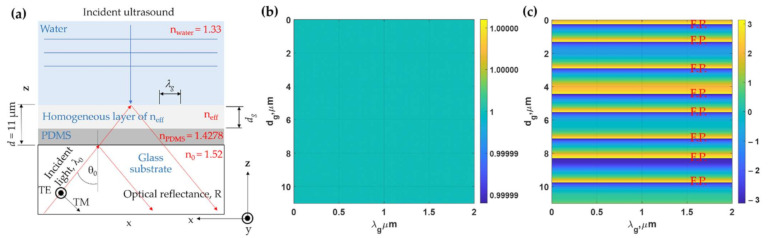
(**a**) Simplified model with homogenous layer and *n_eff_*, (**b**) reflectance of the simplified model, and (**c**) phase of reflection coefficients calculated using RCWA for the simplified model in Figure 8a when illuminated by TM polarized light at 685 nm and the *n*_0_*sinθ*_0_ of 1.37.

Another approach to proving that the horizontal modes are the FP modes excited by the zeroth order of the eigenmodes inside the grating is to treat the grating with a homogeneous layer with the effective refractive index, *n_eff_* [34] as expressed by Equation (3) and depicted in Figure 8a.

(3) $$n_{eff}=FF\;n_{PDMS}+\left(1-FF\right)n_{water}$$

where *n_PDMS_* is the refractive index of PDMS and *n_water_* is the refractive index of water.

**Figure 9:**
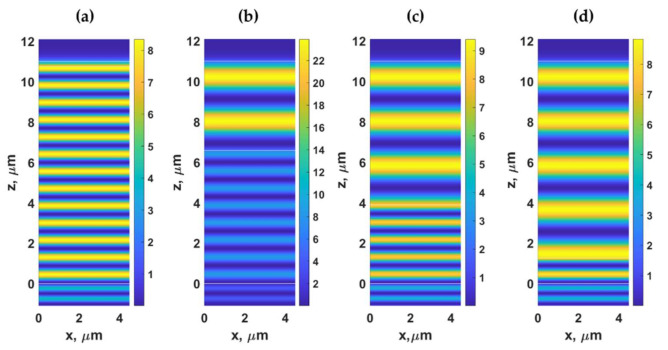
$H_{y}^{2}$ field distribution calculated using RCWA for the simplified models with *n_eff_* calculated with *F.F.* of 0.5, *d* of 11 μm, and (**a**) *d_g_* of 0.24 μm, (**b**) *d_g_* of 4.42 μm, (**c**) *d_g_* of 7.11 μm, and (**d**) *d_g_* of 9.75 μm, when illuminated by TM polarized light at 685 nm and the *n*_0_*sinθ*_0_ of 1.37.

Figure 8b,c shows optical reflectance and the phase response of the reflection coefficients of the homogeneous layer with the effective refractive index calculated from Equation (4). The reflectance of 1 indicates that there is no loss or diffractions to form an intensity dip. However, the phase shown in Figure 8c shows the FP mode positions that agree with the horizontal modes in the grating shown in Figure 5b. Figure 9 shows FP resonances inside the PDMS layer and the effective index layer. The field pattern of the FP modes agrees with the field patterns shown in Figure 6 and Figure 7. The difference between the field pattern in the grating structures and the homogeneous layer was that there were no standing wave patterns along the x-axis in the homogeneous cases.

Let us now consider the vertical modes in Figure 5; they only present in the gratings. The *λ_g_* positions that gave rise to the modes can satisfy the dielectric waveguide grating condition [29], expressed in Equation (4).

(4) $$\lambda_{g,DWG}=\frac{m\lambda_{0}}{2n_{0}sin\theta_{0}}$$

where *λ_g,DWG_* is the grating period when the dielectric waveguide grating condition (DWG) condition [35] is satisfied. The *m* is the waveguide mode number or the modal number of eigenmodes inside the grating.

**Figure 10:**
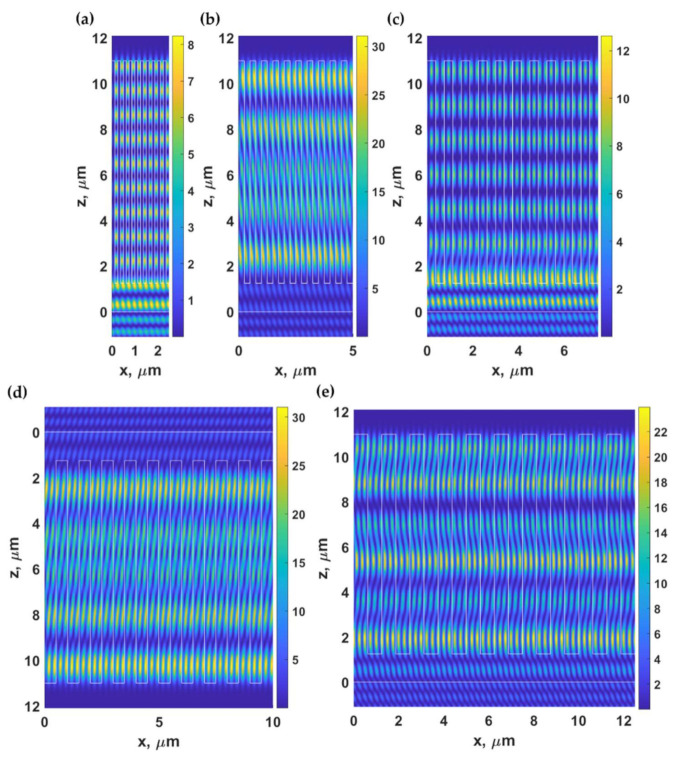
$H_{y}^{2}$ field distribution calculated using RCWA for gratings with (**a**) *λ_g, DWG_* of 0.25 μm (*m of* 1), (**b**) 0.5 μm (*m of* 2), (**c**) 0.75 μm (*m of* 3), (**d**) 1 μm (*m of* 4) and (**e**) 1.25 μm (*m of* 5) with *F.F.* of 0.5, *d* of 11 μm and *d_g_* of 9.75 μm, when illuminated by TM polarized light at 685 nm and the *n*_0_*sinθ*_0_ of 1.37.

For the result shown in Figure 5, the parameters for Equation (4) are the $n_{0}sin\theta_{0}$ of 1.37 and $\lambda_{0}$ of 0.685 μm. These make the right-hand side of Equation (4) equal to 0.25 μm leading to the *λ_g, DWG_* positions of 0.25 μm, 0.5 μm, 0.75 μm, 1 μm, 1.25 μm, 1.5 μm, 1.75 μm, and 2 μm, corresponding to *m* of 1 to 8 respectively. Figure 10 shows $H_{y}^{2}$ field distribution of gratings with the *λ_g, DWG_* of 0.25 μm (*m of* 1), 0.5 μm (*m of* 2), 0.75 μm (*m of* 3), 1 μm (*m of* 4), and 1.25 μm (*m of* 5) with *F.F.* of 0.5, d of 11 μm, and *d_g_* of 9.75 μm when the gratings were illuminated by TM polarized light with the $n_{0}sin\theta_{0}$ of 1.37 and $\lambda_{0}$ of 0.685 μm. Note that the number of standing waves inside one grating period *λ_g, DWG_* is the same as the waveguide mode number *m*.

**Figure 11:**
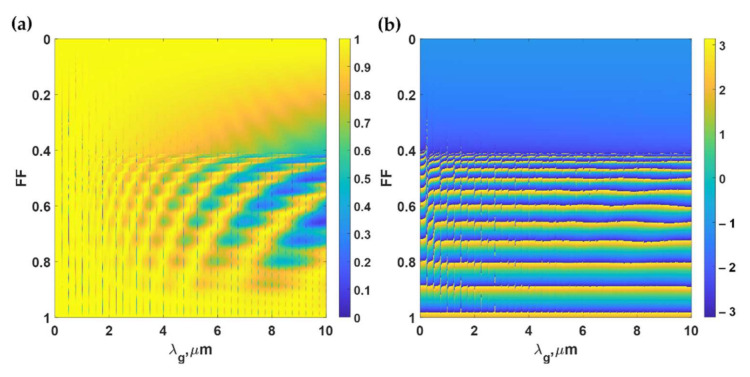
(**a**) Reflectance and (**b**) phase responses in rad of reflection coefficients calculated using RCWA for the PDMS gratings, when the gratings were varied the grating period *λg* from 0 to 10 μm, and the *F.F.* was varied from 0 to 1, and *d* of 11 μm and *d_g_* of 9.75 μm, when illuminated by TM polarized light at 685 nm and the *n*_0_*sinθ*_0_ of 1.37.

Recently, we have reported that the micro-size PDMS grating period can support the FP resonances [23], and the FP dips are present in intensity due to the diffraction mechanism of the PDMS gratings. The strength of the FP coupling can be tuned by the *F.F.*, as shown in Figure 11. A few ways to avoid the FP modes include either (1) avoiding the *F.F.* around 0.4 to 0.9 or (2) designing the PDMS grating with *λ_g, DWG_* below 2 μm.

**Figure 12:**
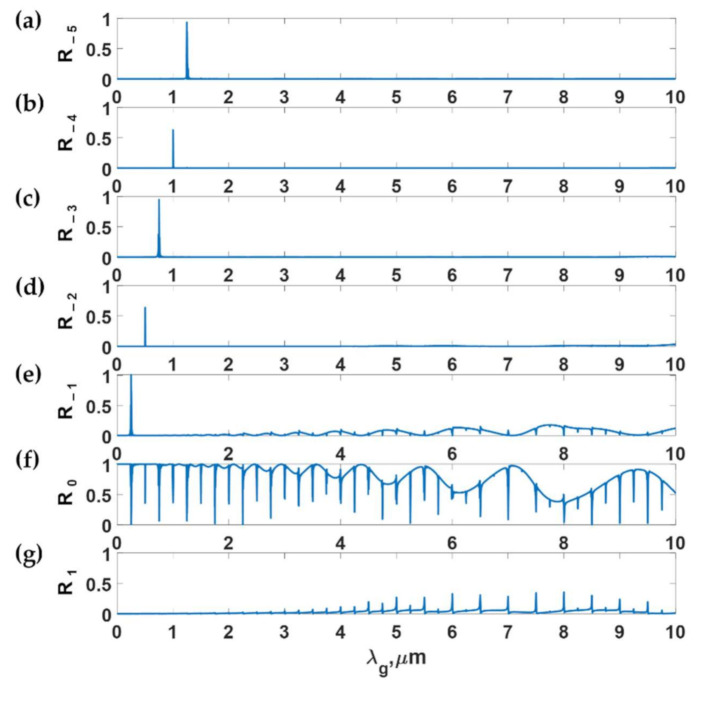
The diffraction efficiencies of (**a**) the −5th order, (**b**) the −4th order, (**c**) the −3rd order, (**d**) the −2nd order, (**e**) the −1st order, (**f**) the 0th order, and (**g**) the 1st order; the efficiencies calculated using RCWA for the gratings varying *λ_g_* from 0 to 10 μm, *F.F.* of 0.5, *d* of 11 μm, and *d_g_* of 9.75 μm when illuminated by TM polarized light at 685 nm and the *n*_0_*sinθ*_0_ of 1.37.

To explain the different loss mechanisms in the FP modes and the DWG modes, let us investigate the intensity in each diffracted order from the gratings with varying *λ_g_* from 0 to 10 μm and the *F.F.* of 0.5, d of 11 μm, and *d_g_* of 9.75 μm. Figure 12a–g shows the diffraction efficiency of the −5th to the 1st diffracted orders. It can be observed that the FP modes dissipate their energy to the −1st and the 1st orders, whereas the DWG modes dissipate their energy through the negative diffracted order corresponding to its DWG mode number *m*. In other words, the incident light is coupled to the *m*^th^ negative diffracted order. This concept has been widely used in narrow-band optical filters [36].

### 3.3. Sensorgram of the PDMS Grating

For the grating with the SAW mode coupling discussed in Section 3.1, the PDMS grating with *λ_g_* of 4 μm, *F.F.* of 0.97, and *d_g_* of 8.87 μm was chosen and quantified for its ultrasonic detection performance using the performance parameters defined in Section 2.3. There is a tradeoff between the *λ_g_* and difficulties in fabrication. One might choose a longer grating period, such as *λ_g_* of 10 μm, which corresponds to the *m^th^* order of 40, indicating that this requires the 40th diffraction order of grating. It then requires the grating edges to be sharp; undercutting or overcutting during the fabrication can degrade the DWG coupling. Therefore, the *λ_g_* of 4 μm, *F.F.* of 0.97, *d* of 11 μm, and *d_g_* of 9.5 μm are well within the limit of two-photon nanoimprinting lithography [37], and the etching linewidth is 120 nm. The aspect ratio of the proposed PDMS grating is less than 3. Recently, Lin et al. [38] have demonstrated the capability of the two-photon nanoimprinting lithography in realizing a high aspect ratio grating of 25.

**Figure 13:**
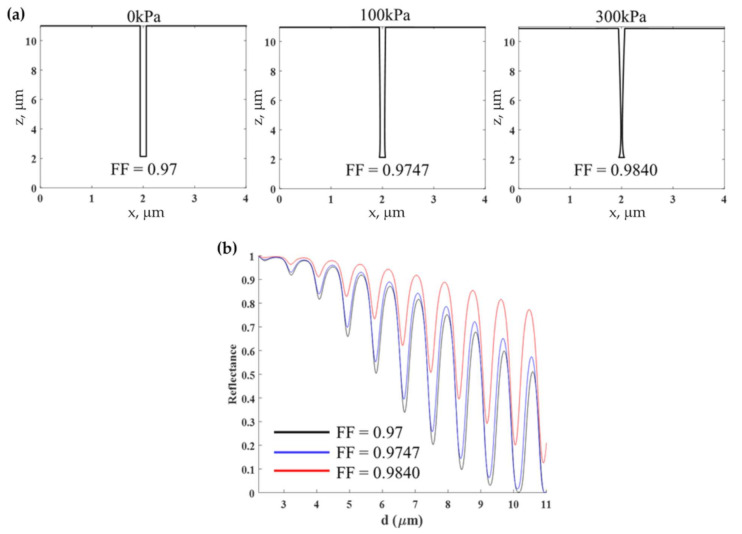
(**a**) Structural deformation when illuminated by different ultrasonic pressures calculated using the FEM, and (**b**) reflectance corresponding to the *F.F.* values in Figure 13a calculated using RCWA for the PDMS grating with *λ_g_* of 4 μm, *F.F.* of 0.97, and *d_g_* of 8.87 μm.

The ultrasound was illuminated on the PDMS grating to the change in the grating geometry. The ultrasound not only compressed the grating thickness in the z-axis but also deformed the shape and the *F.F.* of the grating, as shown in Figure 13. Consequently, the change in *F.F.* can enhance the optical response and the underlining detection mechanism of the PDMS grating. The higher *F.F.* can provide a greater reflectance. The sensorgram for the proposed PDMS grating does not operate on a constant *F.F.* contour, as shown in Figure 13b. Table 1 provides a performance comparison for (1) surface plasmon resonance-based sensor (SPR sensor) [22] with 50 nm uniform gold film [39]; (2) the FP mode in uniform PDMS film [11] with 25 μm PDMS film coated on a glass substrate; (3) the FP mode in uniform PDMS film measured through shearing interferometer [11] with 11 μm PDMS film coated on a glass substrate; (4) the FP mode in bimetallic layer [40] with two gold films of 3.5 nm and 20 nm thicknesses sandwiching a PDMS spacer; (5) the FP mode in Bragg grating [41] with Bragg mirrors consisting of alternating layers of SiO_2_ (n_SiO2_ of 1.4556) and TiO_2_ (n_SiO2_ of 2.5575) sandwiching a PDMS spacer; (6) the FP mode in PDMS dielectric grating [23] with d_g_ of 25 μm, λ_g_ of 112 μm and *F.F.* of 0.5; and (7) the DWG mode in the proposed grating. The responses reported in the Table 1 were the numbers extracted from the references and recalculated using the COMSOL and RCWA to reevaluate the results based on the method described in Section 2.3. The experimental results reported in the references and the calculations agree and are summarized in Table 1.

The SPR has the lowest sensitivity and the second-lowest *FOM* compared to the other structures; however, it is a broadband detector. The FP in the dielectric grating [21] has the highest sensitivity and the second-highest *FOM* compared to other structures. There is a tradeoff between sensitivity, *FOM*, and the detectable range. The DWG mode in the proposed grating has a reasonable sensitivity of 1.08 *×* 10^−6^ Pa^−1^, which is slightly lower than the Bragg reflector’s sensitivity; however, the DWG mode has the highest *FOM* to the other structures without losing too much of the detectable range.

## 4. Conclusions

This paper has provided a theoretical framework to analyze the sensing performance of the sub-wavelength to micron-size PDMS grating period in optical detection of ultrasound. The proposed grating can support both the FP and the DWG modes. The coupling mechanisms and loss mechanisms of both modes have been explained. A simplified model has been proposed to identify and distinguish the mode positions for the FP and the DWG modes based on the effective refractive index theory. The model has provided an insight into the physics of the structure. The proposed grating can enhance the grating’s mechanical and optical properties for quantitative measurement of the incident ultrasonic pressure. The proposed PDMS grating can couple the incident ultrasound to the SAW mode, leading to a significant enhancement in exerting force on the grating surface and the additional thickness compression. When compressed by the ultrasound, the grating did not only change the thickness; it also deformed the *F.F.* This gives rise to an enhancement in the sensitivity and the figure of merit. We have also discussed, quantified, and compared several thin film-based technologies for ultrasonic detection to the proposed PDMS grating. The FOM and detectable ultrasonic pressure range performance of the DWG mode in the proposed grating was better than the FP mode in Bragg mirrors by 2.7 times and 23 times, respectively, without significantly compromising the sensitivity.

## Figures and Tables

**Table 1:** Performance parameters for optical detection of ultrasound for different modes and sensors.

	Sensitivity, Pa^−1^	FOM, Pa^−1^	The Detectable Pressure Range (α), kPa
(1) SPR sensor [22]	6.13 × 10^−9^	4.74 × 10^−8^	Broadband
(2) F.P in uniform PDMS thin film	2.06 × 10^−8^	4.95 × 10^−8^	7130
(3) FP in uniform PDMS thin film with shearing interference [11]	5.10 × 10^−7^	NA	NA
(4) FP in bimetallic layer [40]	6.46 × 10^−8^	7.42 × 10^−7^	2160
(5) FP in Bragg reflector [41]	1.86 × 10^−6^	3.44 × 10^−6^	280
(6) FP in the dielectric grating [23]	1.25 × 10^−5^	1.40 × 10^−5^	67
(7) DWG in the proposed grating	1.08 × 10^−6^	7.83 × 10^−5^	750

## References

[1] Wang L.V. (2017). Photoacoustic Imaging and Spectroscopy.

[2] Berer T., Hochreiner A., Zamiri S., Burgholzer P. (2010). Remote photoacoustic imaging on solid material using a two-wave mixing interferometer. Opt. Lett..

[3] Naetar W., Scherzer O. (2014). Quantitative photoacoustic tomography with piecewise constant material parameters. SIAM J. Imaging Sci..

[4] Beard P. (2011). Biomedical photoacoustic imaging. Interface Focus.

[5] Jeon S., Kim J., Lee D., Baik J.W., Kim C. (2019). Review on practical photoacoustic microscopy. Photoacoustics.

[6] Li X., Yin J., Hu C., Zhou Q., Shung K.K., Chen Z. (2010). High-resolution coregistered intravascular imaging with integrated ultrasound and optical coherence tomography probe. Appl. Phys. Lett..

[7] Shung K.K. (2009). High frequency ultrasonic imaging. J. Med. Ultrasound.

[8] Blanchard F. (2017). High Frequency Ultrasound Generation by Femtosecond Laser Ablation. Proceedings of the Opto-Canada: SPIE Regional Meeting on Optoelectronics, Photonics, and Imaging, Ottawa, ON, Canada, 29 August 2017.

[9] Zhou Q., Lau S., Wu D., Shung K.K. (2011). Piezoelectric films for high frequency ultrasonic transducers in biomedical applications. Prog. Mat. Sci..

[10] Treeby B.E., Zhang E.Z., Thomas A.S., Cox B.T. (2011). Measurement of the ultrasound attenuation and dispersion in whole human blood and its components from 0–70 MHz. Ultrasound Med. Biol..

[11] Learkthanakhachon S., Pechprasarn S., Somekh M.G. (2018). Optical detection of ultrasound by lateral shearing interference of a transparent PDMS thin film. Opt. Lett..

[12] Choi H. (2019). Prelinearized Class-B Power Amplifier for Piezoelectric Transducers and Portable Ultrasound Systems. Sensors.

[13] Chen S.-L. (2017). Review of laser-generated ultrasound transmitters and their applications to all-optical ultrasound transducers and imaging. Appl. Sci..

[14] Kim K.H., Luo W., Zhang C., Tian C., Guo L.J., Wang X., Fan X. (2017). Air-coupled ultrasound detection using capillary-based optical ring resonators. Sci. Rep..

[15] Pongruengkiat W., Pechprasarn S. (2017). Whispering-gallery mode resonators for detecting cancer. Sensors.

[16] Westerveld W.J., Mahmud-Ul-Hasan M., Shnaiderman R., Ntziachristos V., Rottenberg X., Severi S., Rochus V. (2021). Sensitive, small, broadband and scalable optomechanical ultrasound sensor in silicon photonics. Nat. Photonics.

[17] Zhang Z., Dong B., Li H., Zhou F., Zhang H.F., Sun C. (2014). Theoretical and experimental studies of distance dependent response of micro-ring resonator-based ultrasonic detectors for photoacoustic microscopy. J. Appl. Phys..

[18] Beard P.C., Perennes F., Mills T.N. (1999). Transduction mechanisms of the Fabry-Perot polymer film sensing concept for wideband ultrasound detection. IEEE Trans. Ultrason. Ferroelectr. Freq. Control.

[19] Pérez-Cota F., Fuentes-Domínguez R., La Cavera S., Hardiman W., Yao M., Setchfield K., Moradi E., Naznin S., Wright A., Webb K.F. (2020). Picosecond ultrasonics for elasticity-based imaging and characterization of biological cells. J. Appl. Phys..

[20] Shen M., Learkthanakhachon S., Pechprasarn S., Zhang Y., Somekh M.G. (2018). Adjustable microscopic measurement of nanogap waveguide and plasmonic structures. Appl. Opt..

[21] Tadayon M.A., Baylor M.-E., Ashkenazi S. (2014). Polymer waveguide Fabry-Perot resonator for high-frequency ultrasound detection. IEEE Trans. Ultrason. Ferroelectr. Freq. Control.

[22] Sangworasil M., Pechprasarn S., Learkthanakhachon S., Ittipornnuson K., Suvarnaphaet P., Albutt N. Investigation on Feasibility of Using Surface Plasmons Resonance (SPR) Sensor for Ultrasonic Detection: A Novel Optical Detection of Ultrasonic Waves. Proceedings of the 2016 9th Biomedical Engineering International Conference (BMEiCON).

[23] Sukkasem C., Sasivimolkul S., Suvarnaphaet P., Pechprasarn S. (2021). Analysis of Embedded Optical Interferometry in Transparent Elastic Grating for Optical Detection of Ultrasonic Waves. Sensors.

[24] Wangüemert-Pérez J.G., Cheben P., Ortega-Moñux A., Alonso-Ramos C., Pérez-Galacho D., Halir R., Molina-Fernández I., Xu D.-X., Schmid J.H. (2014). Evanescent field waveguide sensing with subwavelength grating structures in silicon-on-insulator. Opt. Lett..

[25] Pechprasarn S., Chow T.W., Somekh M.G. (2018). Application of confocal surface wave microscope to self-calibrated attenuation coefficient measurement by Goos-Hänchen phase shift modulation. Sci. Rep..

[26] Suvarnaphaet P., Pechprasarn S. (2018). Enhancement of long-range surface plasmon excitation, dynamic range and figure of merit using a dielectric resonant cavity. Sensors.

[27] Huttunen T., Malinen M., Kaipio J.P., White P.J., Hynynen K. (2005). A full-wave Helmholtz model for continuous-wave ultrasound transmission. IEEE Trans. Ultrason. Ferroelectr. Freq. Control.

[28] Bruus H. (2012). Acoustofluidics 2: Perturbation theory and ultrasound resonance modes. Lab Chip.

[29] Russell P.S.J. (1986). Optics of Floquet-Bloch waves in dielectric gratings. Appl. Phys. B.

[30] Gaylord T.K., Moharam M. (1985). Analysis and applications of optical diffraction by gratings. Proc. IEEE.

[31] Johansson L., Enlund J., Johansson S., Katardjiev I., Yantchev V. (2012). Surface acoustic wave induced particle manipulation in a PDMS channel—principle concepts for continuous flow applications. Biomed. Microdevices.

[32] Jensen J.A. (2007). Medical ultrasound imaging. Prog. Biophys. Mol. Biol..

[33] Pechprasarn S., Learkthanakhachon S., Zheng G., Shen H., Lei D.Y., Somekh M.G. (2016). Grating-coupled Otto configuration for hybridized surface phonon polariton excitation for local refractive index sensitivity enhancement. Opt. Express.

[34] Sasivimolkul S., Pechprasarn S., Somekh M.G. (2021). Analysis of open grating based Fabry-Pérot resonance structures with potential applications for ultrasensitive refractive index sensing. IEEE Sens. J..

[35] Kazanskiy N.L., Khonina S.N., Butt M.A. (2020). Subwavelength grating double slot waveguide racetrack ring resonator for refractive index sensing application. Sensors.

[36] Horie Y., Arbabi A., Han S., Faraon A. (2015). High resolution on-chip optical filter array based on double subwavelength grating reflectors. Opt. Express.

[37] Huang Z., Tsui G.C.-P., Deng Y., Tang C.-Y. (2020). Two-photon polymerization nanolithography technology for fabrication of stimulus-responsive micro/nano-structures for biomedical applications. Nanotechnol. Rev..

[38] Lin Y., Gao C., Gritsenko D., Zhou R., Xu J. (2018). Soft lithography based on photolithography and two-photon polymerization. Microfluid. Nanofluid..

[39] Somekh M.G., Pechprasarn S. (2017). Surface Plasmon, Surface Wave, and Enhanced Evanescent Wave Microscopy. Handbook of Photonics for Biomedical Engineering.

[40] Wang J., Wang M., Xu J., Peng L., Yang M., Xia M., Jiang D. (2014). Underwater blast wave pressure sensor based on polymer film fiber Fabry–Perot cavity. Appl. Opt..

[41] Buchmann J., Zhang E., Scharfenorth C., Spannekrebs B., Villringer C., Laufer J. (2016). Evaluation of Fabry-Perot Polymer Film Sensors Made Using Hard Dielectric Mirror Deposition. Proceedings of the SPIE BiOS 2016, San Francisco, CA, USA, 13–14 February 2016.

